# Understanding Human Physiological Limitations and Societal Pressures in Favor of Overeating Helps to Avoid Obesity

**DOI:** 10.3390/nu11020227

**Published:** 2019-01-22

**Authors:** Katarina T. Borer

**Affiliations:** Exercise Endocrinology Laboratory, School of Kinesiology, The University of Michigan, Ann Arbor, MI 48109-2013, USA; katarina@umich.edu

**Keywords:** obesity, morbidities, obesifying environment, weight loss, palatability, food intake, nutrient selection, timing of meals and physical activity

## Abstract

Fat gain in our United States (US) environment of over-abundant, convenient, and palatable food is associated with hypertension, cardiovascular disease, diabetes, and increased mortality. Fuller understanding of physiological and environmental challenges to healthy weight maintenance could help prevent these morbidities. Human physiological limitations that permit development of obesity include a predilection to overeat palatable diets, inability to directly detect energy eaten or expended, a large capacity for fat storage, and the difficulty of losing body fat. Innate defenses resisting fat loss include reduced resting metabolism, increased hunger, and high insulin sensitivity, promoting a regain of fat, glycogen, and lean mass. Environmental challenges include readily available and heavily advertised palatable foods, policies and practices that make them abundant, less-than-ideal recommendations regarding national dietary macronutrient intake, and a frequently sedentary lifestyle. After gaining excess fat, some metabolic burdens can be mitigated though thoughtful selection of nutrients. Reduced dietary salt helps lower hypertension, less dietary sugar lowers risk of cardiovascular disease and obesity, and reducing proportion of dietary carbohydrates lowers post-meal insulin secretion and insulin resistance. Food intake and exercise should also be considered thoughtfully, as exercise in a fasted state and before the meals raises glucose intolerance, while exercising shortly after eating lowers it. In summary, we cannot directly detect energy eaten or expended, we have a genetic predisposition to eat palatable diets even when not hungry, and we have a large capacity for fat storage and a difficult time permanently losing excess fat. Understanding this empowers individuals to avoid overeating and helps them avoid obesity.

## 1. Introduction

This review examines human physiological predisposition to overeat, the mechanism thwarting weight loss, and social and environmental factors that led to the sustained rise of obesity and associated metabolic morbidities worldwide, with a particular focus on the United States of America (USA). Some features of human physiology contribute to the ease of gaining fat, such as human opportunistic predisposition to overeat palatable foods even when not hungry and the inability to directly detect energy eaten or expended through exercise. Other features contributing to obesity are the large capacity to store fat compared to non-human primates, and the vigorous metabolic, hormonal, and behavioral obstacles that restrain the loss of accumulated body fat. In addition, social pressures contributing to the ease of overeating include governmental food-availability policies, questionable institutional recommendations for nutrient selection, and advertising by food and restaurant industries that also provide convenient, maximally palatable, and often unhealthy food. The prevention and the reversal of obesity require awareness of the physiological limitations leading to human overeating and a presence of strong defenses against weight loss. As we cannot directly track calories eaten or expended, using sensations of fullness is our partial and limited guide against excessive food consumption. Several examples are provided here on how to limit or counteract a predisposition to seek out nutrients that, in excess, are detrimental, and to select nutrients that, sometimes contrary to public advice, are actually beneficial. Since our feeding mechanism is opportunistic rather than homeostatically regulated, the proposed solution in this review to prevention and reversal of obesity is to approach meals and nutrient intake with an understanding of the ease of overeating, the powerful mechanism resisting weight loss, and limited effectiveness of exercise-associated energy expenditure to counteract it.

The central thesis is presented in a series of five arguments: (1) obesity has serious health and economic consequences and is predominantly driven by overconsumption of food rather than by inadequate contribution of energy expenditure through physical activity; (2) overeating is primarily based on human predisposition for overconsumption of palatable foods, aided by governmental food-supply policies, less-than-ideal institutional recommendations for macronutrient intake, and commercial pressures for the sale of palatable food; (3) the key problem facilitating overeating is human physiological inability to directly detect energy eaten or expended through physical activity; (4) human evolution endowed humans, as opposed to non-human primates, with an impressive capacity for fat storage, and human physiology provides strong metabolic, endocrine, and psychophysical defenses against losses of body mass and stored energy regardless of the starting level of fatness; and (5) an understanding of human physiological limitations permissive to overeating, and of difficulties against reducing body fat levels, exacerbated by societal pressures that facilitate overeating, represent a solution toward prudent selection of quantity and quality of nutrients to prevent obesity or mitigate some of its adverse effects.

## 2. Sustained Rise of Obesity Is Associated with Pathology and Economic Burdens

Obesity in adults, defined as body mass index greater than 30 kg/m^2^, rose rapidly in all of 200 sampled countries around the world during the past 40 years [1]. It increased from 3.2% in 1975 to 10.8% in 2014 in men and from 6.4% to 14.9% in women. Severe obesity is recorded in 2.3% of world’s men and 5% of women, while 0.64% men and 1.6% women attained morbid levels of obesity (body mass index (BMI) greater than 40 kg/m^2^). If these trends continue, by 2025, global obesity will reach 18% in men and 21% in women, and severe obesity will, respectively, surpass 6% and 9%. In the United States (US), 32.2% men and 35.5% of women were obese in 2016 [2]. The estimated annual cost of obesity in the US in 2008 was 147 billion dollars [3], while it was 10.4 billion euros in Europe [4].

The economic and health burdens of obesity are largely a consequence of associated morbidities such as hypertension [5], atherosclerosis [6], hypercoagulability of blood [7], endothelial dysfunction [8], coronary vascular issues [9], kidney dysfunction [10], heart disease [11], stroke [12], and type 2 diabetes (T2D). The close association between obesity, peripheral tissue resistance to insulin action, and T2D is reflected in the frequently used descriptive term “diabesity” [13]. Obesity-associated morbidities increase the risk of death by two- to threefold [14].

**Figure 1:**
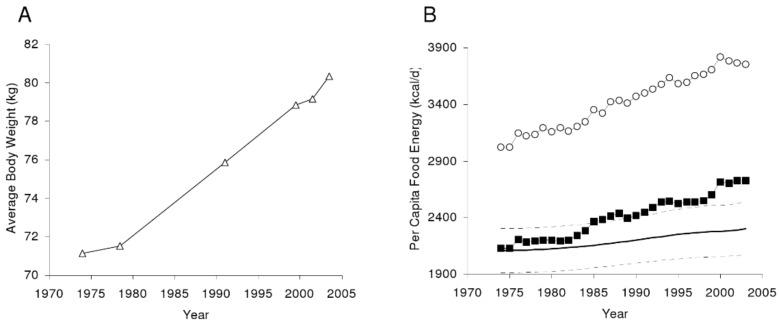
The average United States (US) body weight change as measured by the National Health and Nutrition Examination Survey (**A**). (**B**) Per capita US food availability unadjusted (open circles) and adjusted for wastage. From Hall KD, Guo J, Dore, M, Chow CC. The progressive increase of food waste in America and its environmental impact. PLoS ONE 2009, 4, e7940, doi:10.1371/journal.pone.0007940 [17].

While, from a thermodynamic point of view, the energy loss of physical activity should contribute to body energy balance as much as actual energy consumed, the failure of exercise as electively practiced in developed society to produce substantial body fat loss [15,16] indicates that energy overconsumption [17] is the primary driver of the sustained rise in obesity. At a population level, the average adult US male older than 20 years is estimated to be eating, in recent years, about 862 more kilojoules per day than in 1970s, and the same-age US female, to be eating 1046 kilojoules more (Figure 1).

**Figure 2:**
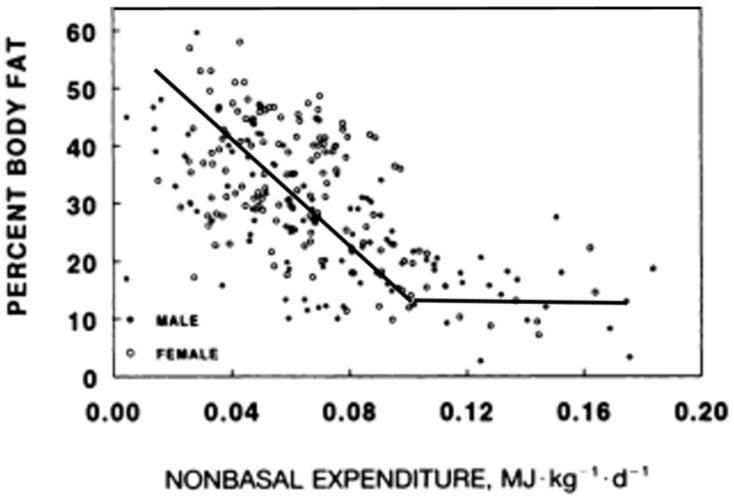
The inverse relationship between metabolic energy expenditure other than resting metabolism and the level of body fat. Modified from Rising, Harper, Fontvielle, Ferraro, Spraul, Ravusin. Determinants of total daily energy expenditure variability in physical activity. Am J Clin Nutr 1994, 59, 800–804, doi:10.1093/ajcn/59.4.800 [21], and Schulz, Schoeller. A compilation of total daily energy expenditure and body weights in healthy adults. Am. J. Clin. Nutr. 1994, 60, 676–681, doi:10.1093/ajcn/60.5.676 [22].

Although relatively intense exercise produces temporary appetite suppression [18], the motivation to be physically active declines in proportion to any increase in obesity in both animals [19] and humans [19,20,21,22] (Figure 2).

The problem being motivation rather than an increase in effort of moving a heavier body was suggested in obese animals engaging in little spontaneous running, but able to run as long and as fast as their lean counterparts on a treadmill equipped with electric shock as a negative reinforcement [22]. Corresponding evidence for the involvement of motivation in the volume of physical activity in humans includes the inverse relationship between voluntary energy expenditure other than metabolism as a function of obesity (Figure 2), in contrast to the temporary great increases in physical activity and food restriction that can produce massive fat losses in response to financial and public incentives of the televised “The Biggest Loser” competition [23,24]. The physiological design to become more energized to move as our body fat declines, and to become more inert as we grow obese may be an unfortunate byproduct of our evolutionary history where increased activity to secure food energy was selected for in situations of food scarcity [25]. Therefore, the solution to prevention and reversal of obesity is to approach meals and nutrient intake armed with an understanding about the ease of overeating and about the limited effectiveness of exercise-associated energy expenditure to counteract it.

## 3. Strong Preference for Intake of Palatable Foods Even When Not Hungry

Even newborn babies smile to sweet tastes and frown to acidic and bitter ones, showing that the predisposition to favor good-tasting nutrients and to reject acidic or bitter ones is inborn [26]. Likewise, adults have neuronal systems in motivational areas of the basal forebrain mediating a drive to eat even unpalatable food when body energy is depleted and to overeat tasty foods even when not hungry [27]. Berridge’s research revealed two areas of the brain mediating hunger and eating for palatability. Brain substrates responsible for motivation to eat when hungry (“wanting” of food reward regardless of its palatability) are in the ventral globus pallidum in the mesolimbic dopamine and opioid motivation circuits. The overlapping hedonic “liking” sites are in the nucleus accumbens in the ventral tegmentum and pallidum, where opioid neurons amplify the incentive salience and sensory pleasure associated with food. A recent study using a number of new techniques in transgenically manipulated mice showed that, in the arcuate hypothalamic nucleus, agouti-related peptide (AgRP) neurons stimulate feeding and can override the pro-opiomelanocortin (POMC) neurons which mediate satiety signals [28].

**Figure 3:**
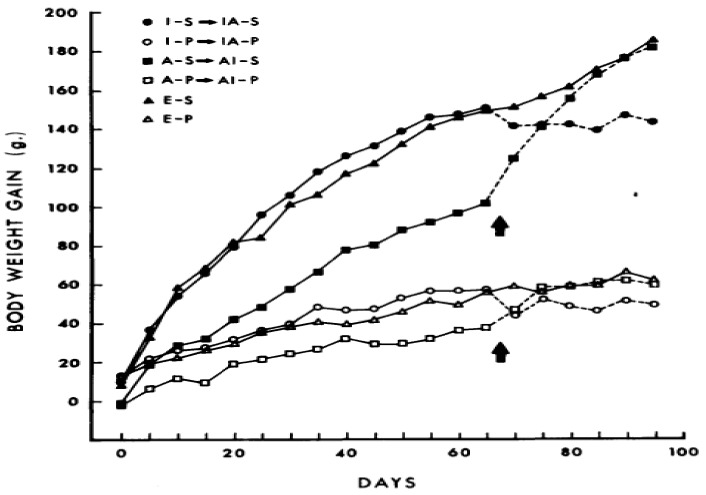
The effect of supplementary palatable “supermarket” food (S) compared to standard Purina chow (P) on weight gain in laboratory rats housed singly (I), in cages allowing exploration and climbing (E), or cages equipped with activity wheels (A). Presence of activity wheels suppressed, but did not eliminate dietary obesity. From Sclafani, Springer. Dietary obesity in adult rats: Similarities to hypothalamic and human obesity syndromes. Physiol. Behav. 1976, 17, 461–471, doi:org/10.1016/0031-9384(76)90109-8, with permission from the publisher [29].

Innate neural circuits providing a positive incentive to overeat palatable food in mammalian omnivores was clearly demonstrated in 1976 [29]. Norway rats were offered palatable and energy-dense “supermarket” diets in addition to standard laboratory chow while housed either in plain cages (I), cages enriched with some opportunities for exploration and climbing (E), or cages with access to activity wheels (A). The effectiveness of palatability of commercial supermarket dietary products (salami, cookies, and chips) in causing significant weight gain is shown in Figure 3, where S compares weight gain when eating supplementary “supermarket” diet option to P, the standard low-fat, high-fiber/carbohydrate Purina chow diet. Exposure to the optional supermarket foods produced striking weight gain and obesity compared to no such effect in rats limited to Purina chow.

Development in these rats of striking dietary obesity from palatable diets was unaffected by an enriched environment and only attenuated by voluntary exposure to activity wheels, whether the activity was available from the start of the experiment or after 65 days of change in dietary exposure. This study in rodents reflects a seemingly similar pattern of weight gain of humans in developed and developing societies, where palatable foods are accessible while physical activity exerts a limited restraining influence.

Social pressures in favor of overeating in developed and developing societies include easy availability of heavily advertised supermarket foods, restaurants that serve large portions of such food (which by itself favors greater food intake [30]), and where the quantity of the food is often combined with low meal prices. Adding to the challenge is the convenience of eating such foods in fast-food establishments and the social policies influencing food availability. In the USA, the implementation of the policy to increase food supply since the 1970s, and in the United Kingdom since the 1980s is also thought to be a significant environmental driver of obesity [30]. The biological predisposition for excessive intake of palatable diets, and the societal facilitation of such intake should further lead to caution in our approach to our dietary intake to avoid excess energy intake that can drive the development of obesity.

## 4. Inability to Detect Energy Eaten or Expended in Physical Activity Represents a Core Human Physiological Handicap for Successful Weight Management

While the massive amount of research into the mechanisms of food intake, hunger, satiation, and specific effects of nutrients on our health is expanding exponentially and focusing on ever finer subcellular details, the larger issue of whether we “regulate” our body composition in adulthood remains controversial and unresolved. At the core of this controversy are alternative hypotheses, one of which posits that human and mammalian body fatness is homeostatically regulated [31] in the way that variables within the internal environment, formulated by Claude Bernard, such as blood glucose, some hormones, and most minerals, maintain their stability. The homeostatic hypothesis was originally stated in terms of hormonal controls as principal drivers of hunger and satiety.

**Figure 4:**
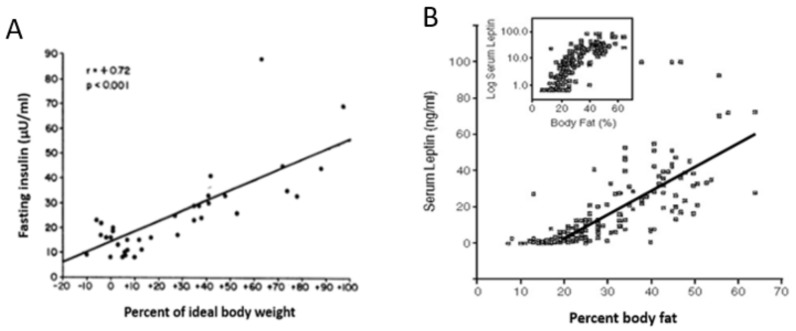
The positive relationship between human ideal body weight (left) or body fat level and basal concentrations of insulin (**A**) and leptin (**B**). Data were modified from Bagdade, Bierman, Porte Jr. The significance of basal insulin levels in the evaluation of the insulin response to glucose in diabetic and nondiabetic subjects. J Clin Invest. 1967, 46(10), 1549–1557 [32] and Considine, Sinha, Heiman, Kriauciunas, Stephens, Nyce, Ohannesian, Marco, McKee, Bauer et al. Serum immunoreactive-leptin concentrations in normal-weight and obese humans. N. Engl. J. Med. 1996, 334, 292–295 [33] respectively.

The hormones insulin and leptin, basal concentrations of which rise in proportion to body fat (Figure 4), were postulated to exert a negative feedback over the ventromedial hypothalamic arcuate nucleus. The feedback was postulated to suppress feeding and increase physical energy expenditure when the fat content in the white adipose tissue (WAT) increased, and to stimulate hunger and reduce physical energy expenditure when the fat content in WAT decreased. The hypothesis appeared to be supported by animal studies in which intracerebral administration of leptin suppressed feeding and caused weight loss, and by the similar efficacy of leptin administration to reduce appetite and obesity in children [34] and rodents [35] rendered obese by the genetic loss of capacity to secrete leptin, but not in animals unable to express leptin receptor [36].

Key tenets of the homeostatic hypothesis were not supported by subsequent studies which did not diminish its widespread acceptance. Evidence not supporting this hypothesis included a trial with obese humans, in whom a wide range of administered leptin concentrations up to pharmacological levels failed to significantly reduce obesity [37]; the ease with which obesity is induced in laboratory animals (and, as discussed in the previous section, also in humans) by energy-rich and/or highly palatable diets despite the concomitant increases in basal leptin and insulin concentrations [38,39]; the well-known endocrine fact that increases in basal insulin and leptin represent a consequence of fat accumulation in WAT cells ([32,33], respectively; Figure 4), and insulin and leptin resistance in WAT and other ectopic tissues [40,41], rather than a cause of compensatory actions to reduce obesity. As shown in Figure 2, obesity, accompanied by increases in basal insulin and leptin, decreases rather than increases spontaneous energy expenditure through physical activity. The final problem is in the claim that relatively minute changes in adiposity caused by daily meals and intermittent bouts of exercise could alter energy regulation by directly affecting hunger and satiety.

**Figure 5:**
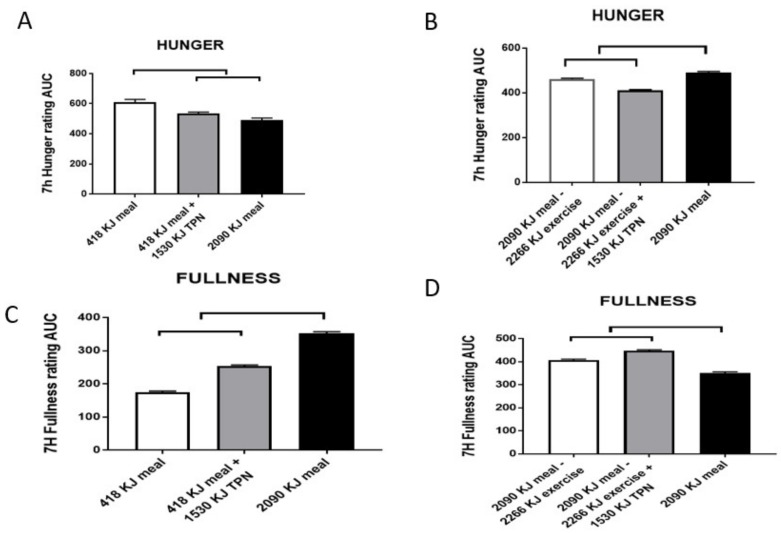
Sensations of hunger (**A**,**B**) and fullness (**C**,**D**) in a study comparing the effects of a 418-kJ breakfast, with or without 1530-kJ intravenous nutrient supplementation (total parenteral nutrition (TPN)) to a 2090-kJ breakfast (AL) (A for hunger and C for fullness respectively), or the energy of a 2090-kJ breakfast reduced by 2266 kJ of exercise energy expenditure (EX), with or without TPN supplementation (B and D respectively). Both hunger and fullness are largely unaffected by TPN, whether the energy shortfall is caused by withholding energy in the morning meal (418 vs. 2090 kJ) or by energy expended from the large meal (2090–2266 kJ). The upper bracket indicates significant differences between the trials (*p* < 0.001). Data are modified from Borer, Wuorinen, Ku, Burant. Appetite responds to changes in meal content, whereas ghrelin, leptin, and insulin track changes in energy availability. J. Clin. Endocrinol. Metab. 2009, 94, 2290–2298, doi:1210/jc.2008-2495 [45].

My alternative hypothesis (e.g., Reference [20]) is that both the intermittent behaviors of feeding and spontaneous physical activity operate in non-homeostatic fashion. The apparent weight stability that prompts the hypothesis of homeostasis results from the contribution of physiological and environmental factors to the energy balance. These consist of habitual physical effort necessary to procure food on one hand, and food consumption necessary to produce fullness within the context of prevailing intermittent meal and activity patterns on the other hand. Possibly the most convincing evidence that the maintenance of stable weight is not a consequence of homeostatic hormonal adjustment of energy eaten or expended is provided in studies demonstrating that humans (and other mammals) lack the capacity to detect and respond to short-term energy exchanges. An early study [42,43] involved feeding volunteers, for 11 weeks, diets of different fat content (20–25% vs. 35–40%) to see whether the study subjects on lower-fat diet would appropriately increase energy consumption to match the higher-energy, 35–40%-fat control diet. There was no change in the volume of food eaten for the duration of the study. This failure to compensate calorically on the low-fat diet resulted in a deficit of 1.22 MJ/day (reported in Reference [42] to correct the original typographical error of 1.22 kJ in Reference [43]) and a weight loss of 2.5 kg in 11 weeks, twice the amount of weight loss on the control diet. A more direct demonstration of human inability to directly consciously detect energy eaten or expended in exercise was provided in more recent studies [44,45], of which the second one [45] measured perception of hunger and fullness, as well as meal-associated changes in plasma leptin and insulin. Subjects were offered a fivefold difference in the energy of the morning meal to be either eaten normally via the orogastric route, by metabolizing calories in the large meal through exercise, or by replacing energy missing in small meals or expended during exercise through intravenous (total parenteral nutrition, TPN) infusion of nutrients. The results were clear. Only the orogastric route allowed conscious detection of the difference in the energy content (and size) of the meals as reflected in the expression of different levels of hunger and fullness (Figure 5). The infusion of parenteral nutrients to correct for the small morning meal or energy expenditure of exercise did not affect either hunger or satiation.

**Figure 6:**
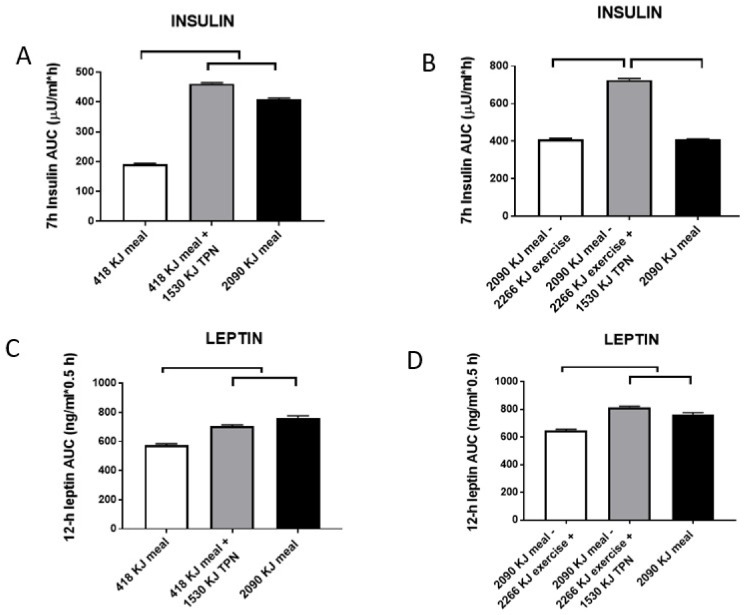
Post-meal responses of insulin (**A**,**B**) and leptin (**C**,**D**) to changes in morning meal size (418 vs. 2090 kJ) and 1530-kJ TPN supplementation while sedentary (**A**,**C**), or after 2266 kJ of exercise energy expenditure (EX) following the 2090-kJ morning meal with or without TPN supplementation (**B**,**D**). Post-meal insulin was highly responsive to changes in both the meal size and TPN energy, but not to exercise energy expenditure. Day-long changes in leptin responded to both differences in the energy provided by two meals and supplemental TPN infusion, and to exercise energy expenditure. Upper and parallel brackets denote significant differences (*p* < 0.001). Data were modified from Reference [45].

On the other hand, both leptin and insulin showed close and proportional responses to energy ingested and infused, or, in the case of leptin, also to energy expended walking on a treadmill (Figure 6).

Together, these two studies allow three conclusions. Firstly, energy intake is not regulated in the short term by detection of energy eaten or expended, but is apparently detected by gastric fullness signals, and likely also endocrine signals from the stomach, proximal intestine and pancreas, responding to the volume and type of nutrients consumed via the orogastric route. At this volumetric and endocrine intake control is working well is also demonstrated by the effectiveness of bariatric surgery in reducing eating and producing weight loss [46]. The second conclusion is that the two metabolic hormones, insulin and leptin, are immediately responsive to energy fluctuations produced by eating, infusion of parenteral nutrients, or, in the case of leptin, also to exercise energy expenditure, but show no consistent relationship with sensations of hunger or fullness. It is likely that changes in the post-meal insulin and leptin concentrations represent primarily metabolic responses, in that the two hormones bear a counter-regulatory relationship [47], and that they both, but particularly leptin, may exert a restraining effect on hunger in the brain over a 24-h period that characterizes leptin responses to caloric fluctuations during the active portion of the day [45,48]. The final conclusion is that short-term fluctuations in leptin concentration in response to meals and exercise could not possibly originate from minute changes in the relatively large WAT tissue mass, but most likely arise from the leptin release from the stomach, which is a second substantial source of this hormone [49,50]. Inability to directly detect energy eaten or expended in physical work is perhaps the most serious cause for thought and caution in approaching eating and weight control. The evidence from the Borer et al. study [45] suggests that immediate postprandial signals of satiation from stomach fullness can distinguish different sizes or volumes of the meal (Figure 5C). That we are capable to respond to stomach stretch alone was elegantly demonstrated in a study [51], in which distension of stomach by infusion of three volumes of liquid into a balloon led to graded ratings of fullness and graded reductions in liquid-diet intake. Upon entering the duodenum, dietary fat and protein elicit secretion of the digestive hormone cholecystokinin (CCK) which meets rigorous criteria of an agent of satiation [52]. CCK sensitizes the stomach to enhance a sensation of fullness [51]. The sensation of fullness should be used as a cue to quantitatively moderate eating when faced with environmental facilitation and innate predisposition toward palatable food. The Japanese, in general, and long-lived inhabitants of the island Okinawa, in particular, practice the Confucian teaching “hara hachi bun me”, of eating until they experience 80% stomach fullness. Occidental societies could benefit by adopting this sensible Oriental cultural practice.

## 5. Human Genetic Predisposition for Obesity and Vigorous Physiological Defenses against Weight Loss Bias Our Physiology toward Fat Gain and Retention

A cursory view of Figure 2 illustrates the stunning levels of human capacity for obesity. It is highly likely that the natural selection toward increased brain size in humans and toward the capacity of women to bear numerous children may have driven the evolution in favor of increased capacity for fat storage. The evolution of larger brains in human ancestors became exponential over the past two million years and resulted in about threefold greater brain mass compared to that in non-human primates [53]. According to the “expensive brain” hypothesis [54], the evolution of high human capacity for obesity is tied to the evolution of human brain size because of the large bioenergetic cost of brain metabolism. Despite the fact that humans have much larger brains per body weight in comparison with other primates or terrestrial mammals, the resting metabolic rate (RMR) for the human body is no greater than that for any other mammal of the same size. The consequence of this paradox is that adult humans allocate a greater proportion (∼20% to 25%) of their daily energy budget to “feed their brains” than other primates. Other primate species allocate only 8% to 10% of daily energy to the brain, and non-primate mammals only 3% to 5% [55]. The capacity to support the bioenergetic needs of brain metabolism was solved through the evolution of the ability to digest animal food sources and fat, reduction in the size of the gastro-intestinal tract (an energy-expensive tissue), and reduction in the proportion of muscle in the body from greater than 44.5% in baboons, bonobos, and chimpanzees, to 41.5%, and corresponding increases in the proportion of body fat [56,57]. This makes humans “under-muscled” and “over fat” compared to other primates [56,57]. Humans also developed the capacity to expand body fat stores in adulthood via adipocyte hyperplasia, as well as hypertrophy [58], making humans among the fattest of mammals [59]. The increased capacity for fat storage is evident even in human infants in their intra-uterine accumulation of fat that reaches 15% of body mass at birth. Human newborns devote about 70% of growth expenditure to fat deposition during the early postnatal months to meet 60% of the daily metabolic need of their relatively large brain [60]. By contrast, newborn baboons have only about 3% body fat [61].

It is also highly likely that the natural selection toward increased brain size and obesity in humans may have driven evolution in favor of capacity to bear numerous children. A large brain and the energetic need to support metabolically expensive pregnancy also led to rapid fat deposition in human females at puberty, subsequent contribution of fat tissue to the energetics of pregnancy and lactation, and reduced sexual dimorphism in human evolution compared to other primate species [56]. Attesting to the evolution and appreciation of obesity in human females is the oldest most famous surviving work of art representing an obviously obese naked woman, Venus of Willendorf, dating from the upper Paleolithic stone age (28,000 to 25,000 years before common era (BCE)). Women are capable of bearing children at intervals of nine to 10 months, as illustrated by the empress Mumtaz Mahal, who gave birth to 14 children in 19 years of marriage to the Mughal shah Jahan who erected the Taj Mahal mausoleum in Agra, India on her behalf. This makes her childbirth rate at 0.74 children/year (slightly lower than the potential of >1), while wild western and mountain gorilla females give birth at a rate of 0.2 infants per year [62], and a similar inter-birth interval between 4.5 and five years in wild chimpanzees [63]. Based on the above data, difference in brain size between humans and non-human primates is paralleled by about two to four times lower incidence of obesity [57] and more than fivefold lower fertility [62,63] in wild non-human primates.

**Figure 7:**
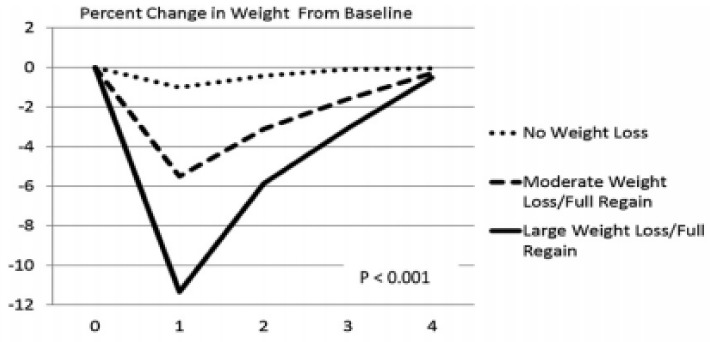
Percentage of post-weight-loss weight change over four years as a function of the magnitude of weight loss. From Wing, Espeland, Clark, Hazuda, Knowler, Pownall, Unick, Wadden, Wagenknecht. Association of weight loss maintenance and weight regain on 4-year changes in CVD risk factors: the action for health in diabetes (Look AHEAD) clinical trial. Diabetes care 2016, 39, 1345–1355, doi:10.2337/dc16-0509, with permission of the publisher [67].

In view of the constellation of morbidities associated with obesity mentioned in Section 1, there were numerous efforts to reverse obesity through dieting and physical energy expenditure. Physiological defenses against weight loss are impressive compared to the ease with which palatability of food, a decline in the motivation for physical activity with increased fatness, and environmental circumstances lead to accumulation of body fat and obesity in humans. Defenses against weight loss have metabolic, hormonal, and behavioral features. A metabolic obstacle to body-fat loss is a reduction in resting metabolic rate that persists even when weight loss is maintained over a long period of time [23,24]. Hormonal defenses are of two kinds. Firstly, the significant reduction in basal leptin concentration after fat loss (Figure 4) almost certainly stems from its reduced secretion from the diminished mass of subcutaneous WAT [64]. A decline in basal leptin concentration lowers this hormone’s central-nervous-system inhibition of hunger drive, which then provides a potent stimulus to eat after the weight loss. Therefore, leptin plays a key role in the control of hunger during negative energy states [65], rather than in the suppression of weight gain as proposed by the homeostatic hypothesis of energy regulation [31], as demonstrated by its ability to suppress hunger in humans who underwent a 10% weight loss [65,66] but not in obese humans [37]. The second important hormonal obstacle to weight loss is the reduction in basal insulin concentration (Figure 4) and a concomitant increase in insulin sensitivity. Since the role of insulin is to promote macronutrient uptake (carbohydrates, amino acids, and lipids) and facilitate their storage in the form of glycogen, tissue protein, and triglycerides, respectively, the health benefits of high insulin sensitivity are mitigated by this hormone’s facilitation of fuel storage and tissue-mass regain. The collective consequence of these metabolic, hormonal, and hunger changes is progressive regaining of deliberately lost weight over months or years (Figure 7 [67]).

The large human capacity for accumulation of body fat, and vigorous metabolic, hormonal, and psychological defenses against fat loss, regardless of the pre-loss fat level, demonstrate that preventing fat gain is more important and effective than efforts to lose it. It should be noted that fat- loss maintenance can be sustained, as reported by the National Weight Control Registry. This organization collects data on adult individuals who maintain a weight loss of at least 14 kg for a year or longer for studies on the successful strategies for weight loss and long-term weight-loss maintenance. The characteristics of successful weight-loss maintenance strategy reported in one of their studies [68] included restriction of total energy intake to about 5648 kJ/day and of fat percentage in the diet to less than 30%, vigorous exercise at least three times per week, and at least once weekly weight monitoring.

## 6. Right Nutrients Matter in Protecting Human Health as Does Timing of Meals and Exercise

Unbiased research and critical and balanced evaluation of the effects of total nutrient intake on our physiology, as discussed above, should be supported and encouraged in view of the general lack of awareness about our inability to track energy in meals or its expenditure through exercise. Similar attention should be paid to the intake of a few specific nutrients that have an impact on the morbidities associated with obesity. Only three examples are mentioned here in view of the massive amount of research in the area of the interactions between nutrition and health. The first two concern our genetically programmed preferences for salty and sweet tastes. The evolution of these two preferences clearly had strong selective advantage, as insufficient intake of salt can be fatal in congenital adrenal hyperplasia due to deficiency of 21-hydroxylase, which can manifest as salt wasting syndrome [69]. Finding sweet nutrient sources supported energy balance in our ancestors and was part of a coevolution of sweetness in fruit that contributed to dispersal of plant seeds eaten by humans and animals. 

The importance of sodium for survival is seen in the presence of duplicate preservation and acquisition mechanisms. Physiological salt preservation depends on the adrenal mineralocorticoid hormone aldosterone, which secures body fluid volume by stimulating sodium reabsorption in the kidney. Sodium is essential for the maintenance of hydration within the extracellular environment. Secretion of aldosterone is controlled by a renin–angiotensin–aldosterone reflex, which is triggered by a decrease in blood pressure sensed by a juxtaglomerular apparatus (JGA) of the kidney. The response is a release of renin by this JGA, which then triggers a hormonal cascade that involves conversion of angiotensin I to angiotensin II and secretion of aldosterone from the adrenal cortex. The second mechanism for securing sodium is the inborn specific hunger and appetite for salt that is responsive to relatively small decreases in plasma sodium precipitated, for example, by sweating [70]. Capitalizing on this innate human preference for salt, food and restaurant industries add too much salt in prepared foods to enhance sales, and this leads to salt overconsumption which is linked to hypertension [71]. Obesity is usually associated with hypertension and often accompanied by salt sensitivity attributable to an inadequate decline in angiotensin II, excessive sodium absorption, and tissue edema [72]. Demonstration of the effectiveness of the low-sodium dietary approaches to stop hypertension (DASH) diet in reducing hypertension justifies efforts to keep daily salt intake below 2.3 g per day.

The innate palatability of sugar displayed even by newborn infants [26] also facilitates overconsumption of this nutrient as it is added to a very large number of commercially available foods. There was a considerable increase in the intake of added dietary sugars from the 1970s to the early 2000s. Although there was a modest decline between 2003 and 2012, in 2012, the US mean adult intake of added sugars was about 1555 kJ or 75 g, and represented 17% of total energy per day [73]. Because a high intake of sugar is linked to obesity and cardiovascular disease, the American Heart Association, in 2009, admonished Americans to eat no more than 25 g of added sugar per day [74].

The third example for caution in nutrient selection involves the health consequences of different proportions of carbohydrates in the human diet. The progression from obesity to prediabetes and type 2 diabetes is characterized by postprandial hyperglycemia and hyperinsulinemia, both markers of carbohydrate intolerance and insulin resistance, where the pancreas secretes excess insulin to move glucose from circulation into insulin-resistant tissues. A recent study assessed to what extent the proportion of carbohydrates in a diet influences insulin resistance, and whether this can be changed by reducing this proportion by half [75]. The question was relevant because of the 2010 recommendation by the Departments of Agriculture and Health and Human Services [76] for Americans to aim for intakes of low dietary fat and for intake of between 45 and 65% of energy in the form of carbohydrates. Since the 1960s and 1970s, when the concern was raised about the role of dietary fat in the elevation of blood cholesterol [77], and about the association between blood cholesterol level, atherosclerosis, and death from coronary heart disease [78], dietary recommendations were widely proffered for Americans to reduce dietary fat and increase dietary carbohydrate intake. The effect in the US was a 30.5% increase in dietary carbohydrate consumption from 213 g per day in 1965 to 278 g per day or 51% of daily energy in 2011 [79]. Concomitantly, there was a rise in US obesity from 3.2% in 1975 to 10.8% in 2014 in men and from 6.4% to 14.9% in women [1]. In the Lin and Borer study [75], subjects were exposed for 24 h to three isocaloric meals containing either 30% or 60% carbohydrate, with or without two hours of moderate-intensity exercise before the last two meals.

**Figure 8:**
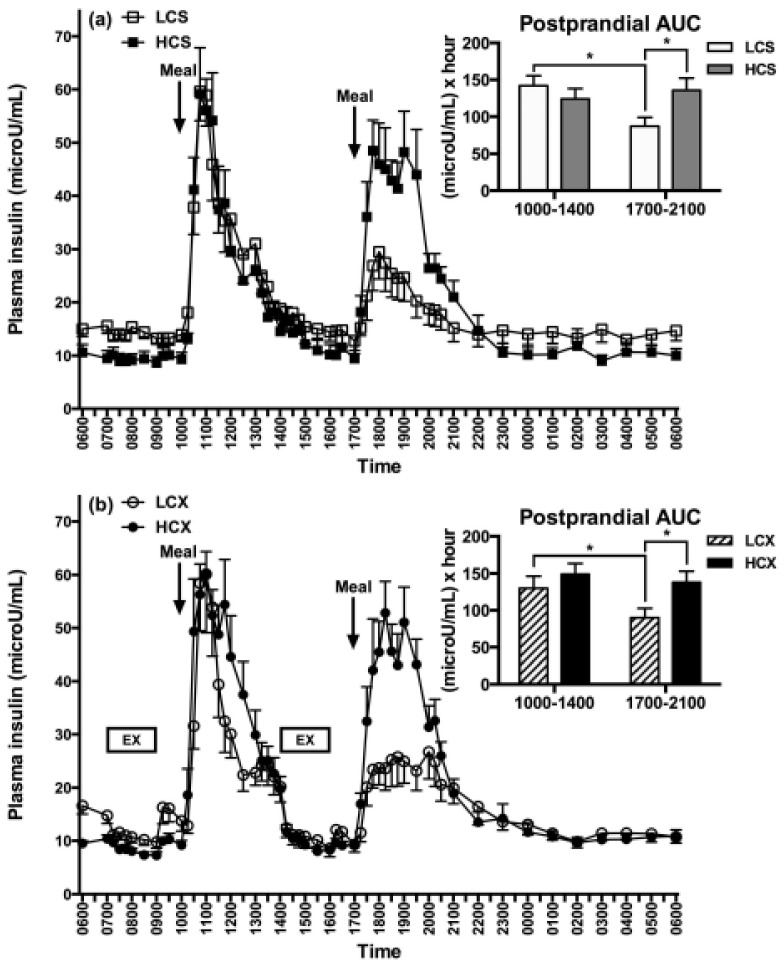
Post-meal plasma insulin responses to low (LCS) and high-carbohydrate meals (HCS) in sedentary (**a**) and exercise (**b**) trials (LCX and HCX, respectively). From Lin, Borer. Third exposure to a reduced carbohydrate meal lowers evening postprandial insulin and GIP responses and HOMA-IR estimate of insulin resistance. PLoS ONE 2016, 11(10): e0165378 [75]. Asterisks indicate significant differences (*p* < 0.05).

**Figure 9:**
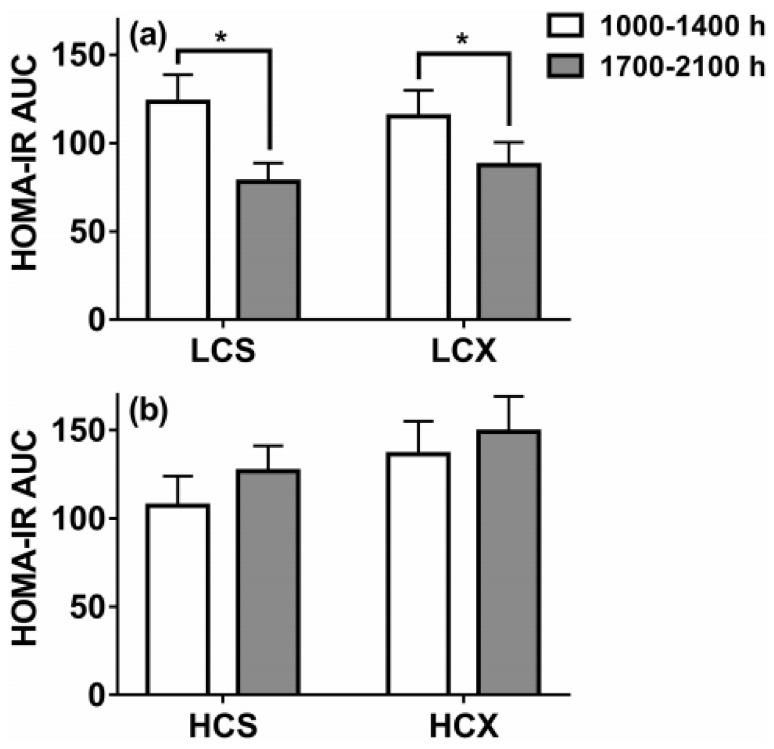
Homeostatic-method measures of insulin resistance based on post-meal insulin and glucose areas under the curve (AUC) after one day of eating low-carbohydrate (30% of energy) or high-carbohydrate (60% of energy) meals while sedentary (LCS and HCS, respectively, (**a**) or after two hours of moderate-intensity pre-meal exercise (LCX and HCX, respectively, (**b**). Data were taken from Reference [75]. Asterisks indicate significant differences (*p* < 0.05).

**Figure 10:**
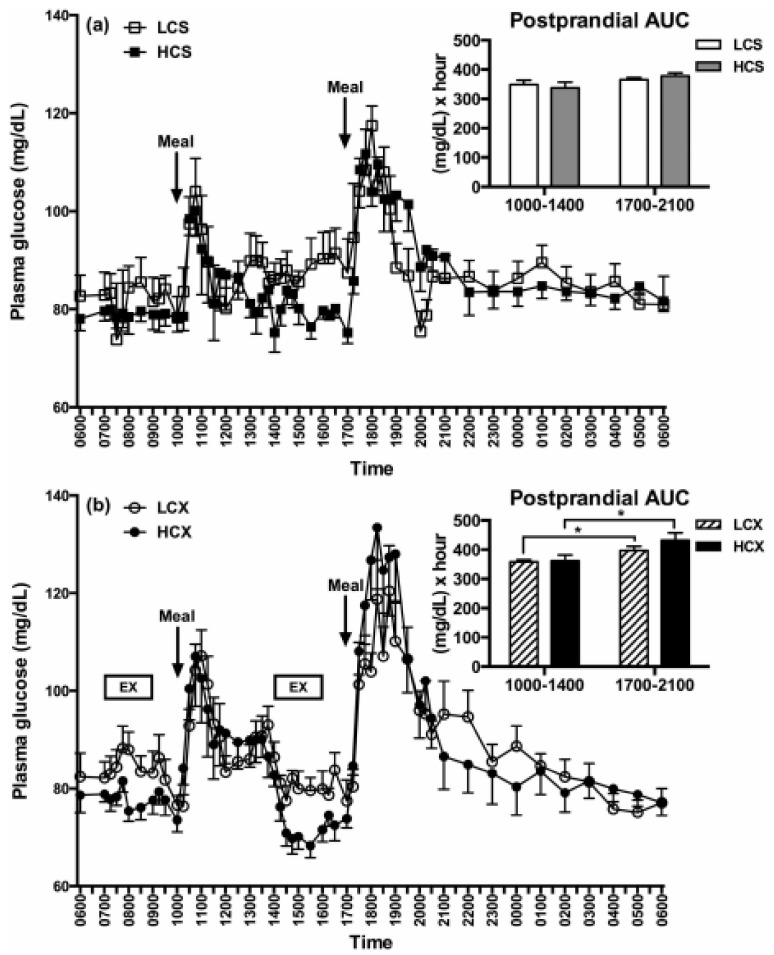
Glucose tolerance as manifested in post-meal changes in plasma glucose AUC after one day of eating low-carbohydrate or high-carbohydrate meals while sedentary (LCS and HCS, respectively, (**a**) or after two hours of moderate-intensity pre-meal exercise (LCX and HCX, respectively, (**b**). Glucose tolerance was unaffected by a difference in dietary carbohydrate, while pre-meal exercise caused glucose intolerance. Data were taken from Reference [75]. Asterisks indicate significant differences (*p* < 0.05).

The third low-carbohydrate meal, but not the high-carbohydrate meal, reduced evening post-meal insulin area under the curve (AUC) by 39% without exercise (Figure 8a), and by 31% after exercise (Figure 8b). This 24-h dietary change reduced evening insulin resistance by 37% without exercise (Figure 9a) and by 24% after exercise (Figure 9b). Pre-meal exercise did not alter postprandial insulin response or the insulin-resistance-lowering effects of a low-carbohydrate diet, but exacerbated evening hyperglycemia (bottom part of Figure 10).

This study suggests that obese and pre-diabetic subjects may, within one day, reduce insulin resistance associated with a high-carbohydrate diet by more than 30% by lowering daily carbohydrate intake to 30% of energy intake. Remarkably, the insulin-lowering and insulin-sensitivity-enhancing effect is a rapid and a purely dietary effect not augmented by the addition of pre-meal exercise.

This study also indicates that timing of exercise and meals is important regarding the variable capacity of exercise to improve postprandial blood glucose response. While exercise before eating in this study elevated post-meal glucose rather than lowering it (Figure 10a), it showed that the timing of meals and exercise needs to be considered. Other studies demonstrated that exercise after meals lowers evening post-prandial glucose and, thus, improves glucose tolerance [80].

## 7. Conclusions

**Figure 11:**
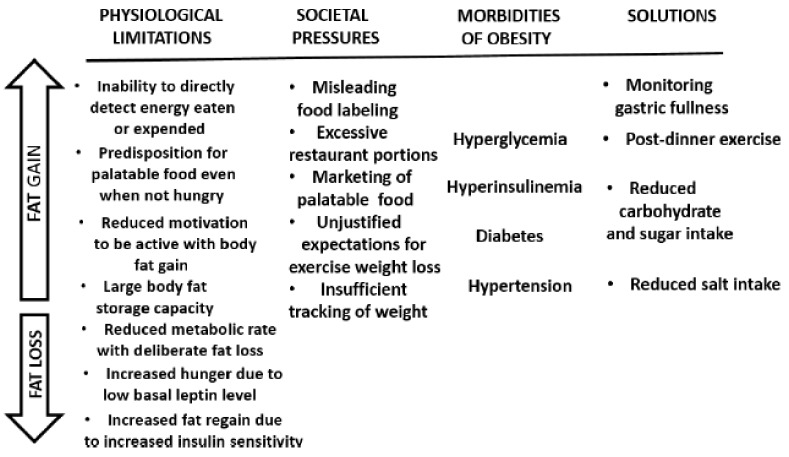
A summary of physiological limitations and some societal pressures facilitating overeating and obesity (upward arrow), and difficulties of losing excess weight (downward arrow). Shown also are some obesity-associated morbidities that can be mitigated by appropriate nutrient intake.

Human evolutionary burdens include a large capacity for fat storage compared to non-human primates, a predilection to overeat palatable diets even when not hungry, a strong preference for sweet and lightly salty foods, and an inability to consciously detect energy eaten or expended. As they gain fat in an environment of over-abundant and convenient “supermarket” food, humans are burdened by the complications of obesity: hypertension, atherosclerosis, hypercoagulability of blood, endothelial dysfunction, coronary vascular issues, kidney and heart disease, and stroke. Societal policies regarding increases in food supply, misguided recommendations regarding the macronutrient proportions in the diet, and commercial interests in marketing highly palatable and often nutrient-poor foods, contribute to overeating in developed and developing societies. These human physiological limitations, some of the societal pressures contributing to overeating that can lead to obesity, and the difficulties of weight loss once excess fat is accumulated are summarized in Figure 11. The figure also identifies some strategies regarding nutrient intake that can mitigate several morbidities that are associated with obesity.

Regrettably, despite the mass of detailed and focused research on human food consumption and on the health effects of diets, there is a general lack of understanding about the physiological limitations leading to easy energy overconsumption that can end in obesity. Ideally, understanding our own genetic vulnerabilities for overeating palatable food even when not hungry, our inability to consciously detect energy eaten or expended, and having a high capacity to store fat and multiple physiological barriers to losing fat should help many avoid getting obese. Should some individuals begin to gain excess fat in the absence of this important understanding, they can use strategic nutrient selections to mitigate some of the metabolic burdens associated with obesity. Reducing dietary salt helps lower hypertension, less dietary sugar lowers risk of cardiovascular disease and obesity, and a smaller proportion of dietary carbohydrates lowers post-meal insulin response and insulin resistance within one day. Food intake and exercise should also be applied thoughtfully, as exercise in a fasted state and before meals raises glucose intolerance, while exercising shortly after eating lowers it. In summary, we cannot directly detect energy eaten or expended, we have a genetic predisposition to eat palatable diets even when not hungry, and we have a large capacity for fat storage and a difficult time permanently losing excess fat. Understanding this empowers individuals to avoid overeating and helps them avoid obesity.
